# Childhood undernutrition in three disadvantaged East African Districts: a multinomial analysis

**DOI:** 10.1186/s12887-019-1482-y

**Published:** 2019-04-23

**Authors:** Kingsley E. Agho, Blessing J. Akombi, Akhi J. Ferdous, Irene Mbugua, Joseph K. Kamara

**Affiliations:** 1 School of Science and Health, Western Sydney University, Locked Bag 1797, Penrith, NSW 2751 Australia; 2 The International Centre for Diarrhoeal Disease Research, Dhaka, Bangladesh; 3 World Vision International, Karen Road, Off Ngong Road, P.O. Box 133, Karen, Nairobi 00502 Kenya; 4 World Vision International, Southern Africa Regional Office, H100, Mbabane, Swaziland

**Keywords:** Morbidity, Mortality, Multinomial, Malnutrition, Undernutrition, East Africa

## Abstract

**Background:**

Undernutrition is an important public health indicator for monitoring nutritional status and survival. In spite of its importance, undernutrition is a significant problem health problem in many East African communities. The aim of this study was to identify factors associated with childhood undernutrition in three disadvantaged East African Districts.

**Methods:**

We examined data for 9270 children aged 0–59 months using cross-sectional survey from Gicumbi District in Rwanda, Kitgum District in Uganda and Kilindi District in Tanzania. We considered the level of undernutrition (stunting, wasting and underweight) as the outcome variables with four ordinal categories (severely undernourished, moderately undernourished, mildly undernourished, and nourished). Generalized linear latent and mixed models (GLLAMM) with the mlogit link and binomial family that adjusted for clustering and sampling weights were used to identify factors associated with undernutrition among children aged 0–59 months in three disadvantaged East African Districts.

**Results:**

After adjusting for potential confounding factors, the odds of a child being stunted were higher in Gicumbi District in Rwanda while the odds of a child being wasted and underweight were higher in Kitgum District in Uganda. Having diarrhoea two weeks prior to the survey was significantly associated with severe undernutrition. Wealth index (least poor household), increasing child’s age, sex of the child (male) and unavailability of water all year were reported to be associated with moderate or severe stunting/wasting. Children of women who did not attend monthly child growth monitoring sessions and children who had Acute Respiratory Infection (ARI) symptoms were significantly associated with moderate or severe underweight.

**Conclusions:**

Findings from our study indicated that having diarrhoea, having ARI, not having water availability all year and not attending monthly child growth monitoring sessions were associated with undernutrition among children aged 0–59 months. Interventions aimed at improving undernutrition in these disadvantaged communities should target all children especially those children from households with poor sanitation practices.

**Electronic supplementary material:**

The online version of this article (10.1186/s12887-019-1482-y) contains supplementary material, which is available to authorized users.

## Background

Undernutrition is a major public health problem and an important health indicator for monitoring nutritional status and survival of children under- 5 years in many developing countries around the world [1]. Measures of childhood undernutrition are used to track development progress and socioeconomic inequalities in many low and middle-income countries. Suboptimal nutrition in the first 1000 days of life could lead to impaired physical development, which has a long-term impact on cognitive ability thus resulting in reduced educational performance and economic productivity in adulthood [2]. Undernutrition lowers immunity thereby predisposing a child to the higher risk of infections, as well as increases the frequency and severity of such infections, and also delay recovery. The relationship between undernutrition and infection creates a vicious cycle of worsening illness and deteriorating nutritional status as undernutrition can make a child more susceptible to infection, and infection also contributes to undernutrition [3].

Sub-Saharan Africa bears one of the highest burdens of undernutrition. In 2016, more than one-third of stunted children (38%) and more than one-quarter of wasted (27%) children lived in sub-Saharan Africa. However, a more detailed look into the distribution of undernutrition within sub-Saharan Africa shows that Eastern Africa (36.7%) has a higher prevalence of stunting compared to Western Africa (21.4%), Central Africa (32.5%), and Southern Africa (28.1%) [4]. While Western Africa (8.5%) has a higher rate of wasting than Central Africa (7.3%), Southern Africa (5.5%), and Eastern Africa (6.5%) [4], these estimates reveal regional disparities in the distribution of undernutrition, thus the need to identify region-specific factors contributing to the distribution of undernutrition across the regions.

The predictors of childhood undernutrition are well-researched and could be classified as either proximate or distal, and affect the nutritional status of children at different levels [5, 6]. Proximate factors operate at the individual and child level which includes age, sex, birth size, birth order, birth interval and infections [7, 8]. The distal factors include a wider range of conceptual factors within the socio-cultural, economic, environmental, climatic and political context which influence food (in) security, sanitation, access to health care services and education at the household and community level [9]. Studies have reported that these factors tend to vary spatially depending on geographic location and climatic conditions. Previous studies have reported a relationship between undernutrition and geographical region, clearly acknowledging that a child’s geographic location is an important modifier of known determinants of undernutrition [5, 7]. However, despite the huge body of evidence on the relationship between undernutrition and geographical region, there is limited evidence of analysis of the factors associated with childhood undernutrition across communities within highly burdened countries in the same sub-region in order to identify the most consistent factors. Given the high rate of undernutrition in Eastern Africa, this study aims to identify the specific factors associated with childhood undernutrition in three East African Districts in order to drive targeted interventions within these communities. With a decrease in undernutrition within communities, there will be a corresponding decrease at the national and sub-regional level thus setting Eastern Africa on the path to achieving the World Health Organization (WHO) global nutrition target by 2025.

## Methods

### Study area

Gicumbi district is situated in the Northern Province of Rwanda and has a population of 395,606 residents. Gicumbi District covers 21 sectors, 109 cells, 630 villages (Imidugudu). It has 23 health centres, 1 district hospital and 1 prison clinic. Rwanda has a health development strategy based on decentralized management and district-level care [10]. Kilindi district is located in the northern zone of Tanzania with a population of 236,833 residents. Kilindi District comprises of 16 rural wards and 102 villages. It has 30 dispensaries, 3 health centres and one hospital [11]. Tanzania has a hierarchical health system made up of the dispensaries found in every village, health centres at the ward level, district hospital at the district level, the regional referral hospital at the regional level, the zone hospitals at the tertiary level and the national hospital at the national level. There are also some specialized hospitals which do not fit directly into this hierarchy and therefore are directly linked to the ministry of health [12]. Kitgum district is located in the northern region of Uganda and has a population of 247,800 residents. It comprises 51 parishes and 437 village councils [13]. Kitgum district has 2 hospitals and 23 health centres; 21 are government owned while 4 are owned by non -government Organizations. Health services delivery in Uganda is decentralized within national, districts and health sub-districts levels with referral hospitals at the national level and health centres at the district and sub-district levels [14].

The sample was in two stages. In the first stage, a total of 20 villages (clusters) were selected from cells for Gicumbi, wards for Kilindi and Parishes for Kitgum. In the second stage, 32 households were randomly selected in each selected villages (clusters). The detailed sampling procedure for Gicumbi in Rwanda has been reported elsewhere [15]. For district-level results, sample weights will be used, and sampling weight was calculated by the product of the reciprocal of the sampling fractions employed in the selection of (cells for Gicumbi, wards for Kilindi and Parishes for Kitgum). For the combined analysis of the three datasets, we re-normalised our sampling weights by computing the total sum of weights for each district and divide each district survey sampling weights with the total sum of weights.

### Data source

Our dataset was obtained from a survey conducted during the harvest period, from 21st– 31st of January, 2016 in Gicumbi district in Rwanda, Kitgum district in Uganda and Kilindi district in Tanzania. The survey was commissioned as part of World Vision Rwanda, Uganda and Tanzania funding service agreement to generate evidence to influence maternal and child health programmes which aimed to reach 36,250 disadvantaged beneficiaries in these East African districts. The Maternal Newborn Child Health (MNCH) Project aimed to collect health and related indicators to identify the health needs of women and children and to establish priorities for evidence-based planning, decision-making in these regions. The program was an opportunity for World Vision to embed knowledge and action of the organisation’s ‘7–11’ interventions for maternal and child survival in the Region [16]. World Vision uses the 7–11 approach to prevent maternal and child mortality and morbidity through 7 key interventions for a mother and 11 interventions for the child. The intervention for the mother are: diet, deworming and iron supplements, prevention of infectious diseases, malaria prevention and treatment, appropriate pregnancy spacing, birth preparedness, and access to antenatal and postnatal maternity services. The 11 interventions for the child are appropriate breastfeeding, newborn care, timely complementary feeding, age-appropriate immunisation, sufficient iron intake, consistent hand washing prevention and treatment for acute malnutrition, prevention and treatment of malaria, and acute respiratory infection. Others are timely administration of oral rehydration therapy to treat diarrhoea, prevention and care for pediatric Human Immunodeficiency Virus (HIV), and timely deworming [17].

### Study outcomes

The nutritional status of children under five years of age was measured anthropometrically. We considered height-for-age (stunting), weight-for-height (wasting) and weight-for-age (underweight). The height-for-age index is an indicator of linear growth retardation and cumulative growth deficits in children, Weight-for-height index measures body mass in relation to height and reflects the current nutritional status of the child. Weight-for-age takes into account both acute malnutrition (wasting) and chronic malnutrition (stunting), but it does not distinguish between stunting and wasting. The index is calculated using growth standards published by WHO in 2006. These growth standards were generated through data collected in the WHO Multicentre Growth Reference Study and expressed in standard deviation units from the Multicentre Growth Reference Study median [18]. Child undernutrition status was categorized into four categories - severe undernutrition (< − 3.0 Z-score), moderate undernutrition (− 3.0 to − 2.0 Z-score) and mild undernutrition (− 2.0 to − 1.0 Z-score) and proper nutrition (≥ − 1.0 Z-score). The level of childhood undernutrition (stunting, wasting and underweight) was considered as the outcome variables with four ordinal categories (severely undernourished, moderately undernourished, mildly undernourished, and nourished).

### Potential confounders

The potential confounding factors were organised into four distinct groups: Socio-economic and demographic (Districts, primary caregiver, education level, marital status, household wealth index and food security); child (fever in two weeks preceding each survey, acute respiratory infection (ARI) in two weeks prior to each survey, diarrhoea in the two weeks preceding each survey, sex of baby and child’s age in months); maternal and child health (antenatal care and attended child monthly growth monitoring sessions); health services and environmental factors (quality of care from health services, place of delivery, water available all year, sources of drinking water and type of toilet facility).

The household wealth index variable measures basic household needs for all children 5–18 years. The household wealth index was constructed by assigning weights to three basic household needs for children 5–18 years (i.e. difficulty providing at least two sets of clothes for all children aged 5–18 years living in the household, difficulty providing a pair of shoes for all children aged 5–18 years living in the household and difficulty paying school fees or school contribution for all children aged 5–18 years living in the household) using principal components analysis. The household wealth index was divided into three categories: poorest, middle and least poor [19]. Improved and unimproved sources of drinking water and type of toilet facility were categorised based on the WHO and UNICEF Joint Monitoring Programme guidelines [20]. Birth order and number of children per household were not collected. Higher birth order and number of children per household may result in higher susceptibility to undernutrition due to impact on income.

### Statistical analysis

Re-normalised weight was used for Survey (SVY) tabulation that adjusts for clustering, and sampling weights were used to determine the percentage and frequency count of all selected characteristics and, district-specific weights were used for the Taylor series linearization method in the surveys when estimating 95% confidence intervals around prevalence estimates of undernutrition by severity in each district.

Multivariate multinomial logistic regression model guided by the conceptual framework was used to determine factors associated with childhood undernutrition (stunting, wasting and underweight) with nourished children used as reference category.

In the multivariate analyses, a four-stage model was carried out, in the first stage model, socio-economic and demographic factors were entered into the model, and a stepwise backward elimination method was used to remove the non-significant factors (*p* > 0.05). In the second stage model, the significant factors in the first stage model were added to the child level factors, and this was followed by another stepwise backward elimination procedure which retained all the significant factors. A similar procedure was employed for the third stage model which included the individual (maternal and child) level factors as well as health services factors and the final stage model which introduced environmental factors. After completion of all four modelling stages, the factors that were significantly associated with the outcomes were retained. All statistical analyses were conducted using STATA/MP Version.14.1 (StataCorp, College Station, Texas, USA) and adjusted odds ratios (AORs) and their 95% confidence intervals (CIs) obtained from the adjusted multivariate multinomial logistic regression model were used to measure the factors associated with childhood undernutrition.

## Results

Distribution of children aged 0–59 months in three East African Districts is presented in Table 1. The majority of women were from Kitgum district in Uganda, and the proportion of respondents who were educated up to secondary level was about 9%. Women who were never married made up 57% while 89% of the women reported being the primary caregiver.

**Table 1:** Distribution of children aged 0–59 months in three disadvantaged East African Districts (*n* = 9270)

Variables	n	Percent (%)
Socio-economic and demographic factors		
District (Country)		
Gicumbi (Rwanda)	2349	25.3
Kitgum (Uganda)	4267	46.0
Kilindi (Tanzania)	2654	28.6
Primary caregiver		
Mother	8261	89.1
Others	1009	10.9
Education level (*n* = 9266)		
No schooling	3864	41.7
Primary	4590	49.5
Secondary and Tertiary	812	8.8
Marital status (*n* = 9266)		
Never married	5268	56.8
Currently married	3789	40.9
Formerly married	209	2.3
Household wealth index		
Poorest	4744	51.2
Middle	2713	29.3
Least Poor	1813	19.6
Child factors		
Sex of child		
Male	4552	49.1
Female	4718	50.9
Child’s Age in months		
0–23 months	6295	67.9
24–59 months	2975	32.1
Maternal and child’s health factors		
Antenatal care (ANC, *n* = 8904)		
Inadequate (< 4 visits)	2374	25.6
Adequate (4+ visits)	6530	70.4
Attended child monthly growth monitoring sessions (*n* = 8163)		
Yes	6941	74.9
No	1722	18.6
Health services and Environmental factors		
Quality of care from health services (*n* = 8170)		
Very good	1379	14.9
Good	4857	52.4
Not good	1934	20.9
Place of delivery (*n* = 9266)		
Government health unit	7003	75.5
Others	2263	24.4
Water availability all year		
Yes	7071	76.3
No	2199	23.7
Sources of drinking water		
Improved	5954	64.2
Unimproved	3316	35.8
Type of toilet facility		
Improved	469	5.1
Unimproved	8801	94.9
Household food security		
Food security	1494	16.1
Mild	361	3.9
Moderate	3950	42.6
Food insecurity	3465	37.4
Fever		
No Fever	6003	64.8
Had Fever	3267	35.2
Diarrhoea		
No diarrhoea	7802	84.2
Had diarrhoea	1468	15.8
ARI		
No ARI	7786	84.0
Had ARI	1484	16.0
Child undernutrition status		
Stunting (*n* = 8706)		
Not stunted	3516	37.9
Mildly stunted	2405	25.1
Moderately stunted	1631	16.8
Severely stunted	1154	11.8
Wasting (*n* = 8706)		
Not wasted	6067	65.4
Mildly wasted	1917	20.7
Moderately wasted	572	6.2
Severely wasted	150	1.6
Underweight (*n* = 8706)		
Not underweight	7264	70.4
Mildly underweight	985	10.6
Moderately underweight	321	3.5
Severely underweight	138	1.5

Acute respiratory infection (ARI)

Weighted total = 9270 unless otherwise given in parenthesis

The proportion of male and female children was relatively constant, and 51.2% of respondents were from poorest households. Over two-thirds of women reported having water availability all year and over 80% had no diarrhoea or ARI. The proportion of mildly, moderately and severely stunted were 25.1, 16.8 and 11.8%, respectively while a high proportion of children reported either not wasted or not underweight. Percentage distribution of childhood undernutrition aggregated by three East African Districts was reported Additional file 1: Table S1.

### Prevalence of undernutrition

**Fig. 1:**
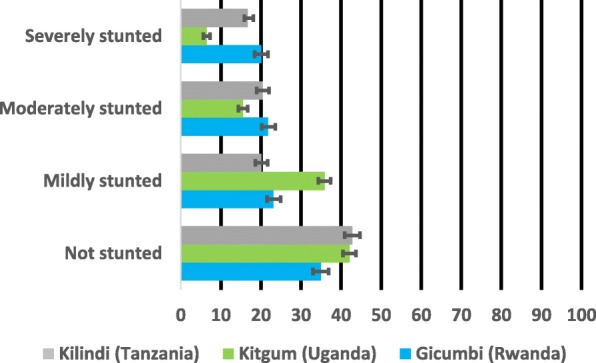
Prevalence and 95% confidence intervals (CIs) of stunting by severity

**Fig. 2:**
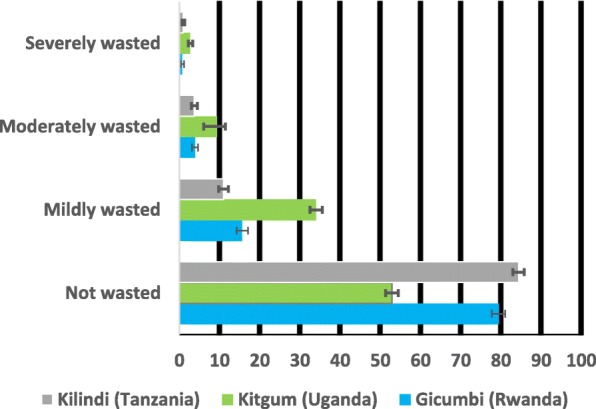
Prevalence and 95% confidence intervals (CIs) of wasting by severity

**Fig. 3:**
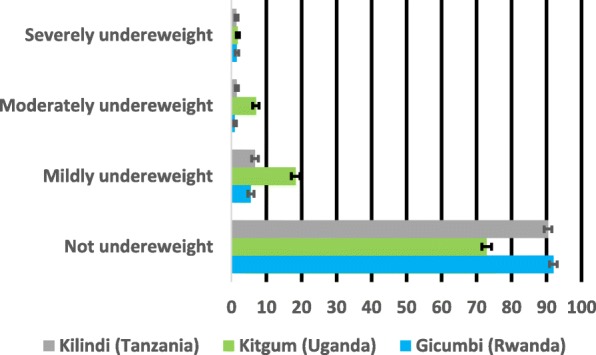
Prevalence and 95% confidence intervals (CIs) of underweight by severity

Figures 1, 2 and 3 show the prevalence and 95% confidence Intervals of undernutrition (stunting, wasting and underweight) by severity. In the figures, if the 95% confidence intervals overlap implies non-significant different at the 95% confidence level. In Fig. 1, the prevalence of severe and moderate stunting was significantly higher in Gicumbi district and Kilindi district but lower in Kitgum district while the prevalence of mild stunting was higher in Kitgum district compared with the other two districts (Gicumbi and Kilindi).

In Fig. 2, the prevalence of mild, moderate and severe wasting was significantly higher in Kitgum district compared with Gicumbi and Kilindi districts while the prevalence of underweight was also higher in Kitgum district than the other two districts (Gicumbi and Kilindi) as represented in Fig. 3.

### Factors associated with mild, moderate and severe stunting

Table 2 shows the factors associated with mild, moderate and severe stunting in children aged 0–59 months in three disadvantaged East African districts. The odds of a child being moderately or severely stunted significantly decreased in Kitgum and Kilindi districts compared to Gicumbi district. Significantly increased odds of mild, moderate and severe stunting were reported among household wealth index (least poor), primary caregiver (mothers), male child, child aged 24–59 months and children who reported having diarrhoea.

**Table 2:** Factors associated with mildly, moderately and severely stunted in children aged 0–59 months in three disadvantaged East African Districts

Variables	Mildly stunted		Moderately Stunted		Severely stunted	
	AOR (95%CI)	*P*-value	AOR (95%CI)	P-value	AOR (95%CI)	*P*-value
District (Country)						
Gicumbi (Rwanda)	1.00		1.00		1.00	
Kitgum (Uganda)	1.10 (0.94,1.29)	0.227	0.47 (0.40,0.57)	< 0.001	0.18 (0.15,0.23)	< 0.001
Kilindi (Tanzania)	0.92 (0.76,1.10)	0.342	0.93 (0.77,1.1)	0.495	0.67 (0.52,0.82)	< 0.001
Household wealth index						
Poorest	1.00		1.00		1.00	
Middle	1.09 (0.95,1.26)	0.209	1.07 (0.91,1.26)	0.397	1.04 (0.86,1.26)	0.679
Least Poor	1.37 (1.18,1.59)	< 0.001	1.30 (1.09,1.5)	0.003	1.36 (1.11,1.67)	0.003
Primary caregiver						
Mother	1.00		1.00		1.00	
Others	0.85 (0.72,1.00)	0.056	0.73 (0.59,0.91)	0.005	0.45 (0.32,0.64)	< 0.001
Sex of the baby						
Male	1.00		1.00		1.00	
Female	0.81 (0.73,0.90)	< 0.001	0.69 (0.61,0.78)	< 0.001	0.54 (0.47,0.62)	< 0.001
Child’s Age in months						
0–23 months	1.00		1.00		1.00	
24–59 months	2.18 (1.91,2.49)	< 0.001	2.53 (2.16,2.97)	< 0.001	2.45 (2.03,2.96)	< 0.001
Diarrhoea						
No diarrhoea	1.00		1.00		1.00	
Had diarrhoea	1.13 (0.97,1.31)	0.126	1.28 (1.07,1.53)	0.008	1.80 (1.45,2.33)	< 0.001

Confounding factors adjusted for are: Socio-economic and demographic factors; child factors; child undernutrition status, maternal, health services and environmental factors and child’s health factors

### Factors associated with mild, moderate and severe wasting

Table 3 shows the factors associated with mild, moderate and severe wasting in children aged 0–59 months in three disadvantaged East African districts. Mild, moderate and severe wasting significantly increased in Kitgum district compared with Kilindi and Gicumbi districts. Significantly increased odds of mild and severe wasting were observed among the middle household wealth index while significantly increased odds of moderate and severe wasting were reported among the least poor. Maternal education (no schooling), primary giver (mothers), male child, no water availability all year and children who reported having diarrhoea were significantly associated with mild, moderate and severe wasting. The odds of moderate and severe wasting were significantly higher in children aged 24–59 months while the source of drinking water (unimproved) was significantly associated with mild wasting.

**Table 3:** Factors associated with mildly, moderately and severely wasted in children aged 0–59 months in three disadvantaged East African Districts

Variables	Mildly wasted		Moderately wasted		Severely wasted	
	AOR (95%CI)	*P*-value	AOR (95%CI)	*P*-value	AOR (95%CI)	*P*-value
District (Country)						
Gicumbi (Rwanda)	1		1		1	
Kitgum (Uganda)	3.90 (3.28, 4.65)	< 0.001	2.99 (2.22, 4.04)	< 0.001	1.94 (1.11, 3.39)	0.021
Kilindi (Tanzania)	0.66 (0.54, 0.80)	< 0.001	0.96 (0.68, 1.36)	0.824	0.46 (0.2, 0.89)	0.022
Maternal education level						
No schooling	1		1		1	
Primary	0.91 (0.81, 1.02)	0.096	0.91 (0.81, 1.02)	0.346	0.70 (0.9, 0.99)	0.043
Secondary+	0.99 (0.82, 1.21)	0.970	0.75 (0.53, 1.06)	0.101	0.77 (0.43, 1.41)	0.410
Household wealth index						
Poorest	1		1		1	
Middle	1.25 (1.08, 1.44)	0.002	1.02 (0.80, 1.29)	0.900	2.98 (1.96, 4.53)	< 0.001
Least Poor	1.02 (0.88, 1.18)	0.816	1.43 (1.14, 1.79)	0.002	1.59 (1.00, 2.53)	0.048
Primary caregiver						
Mother	1		1		1	
Others	0.96 (0.81, 1.14)	0.651	1.00 (0.77, 1.30)	0.992	0.36 (0.19, 0.67)	0.001
Sex of the baby						
Male	1		1		1	
Female	0.86 (0.77, 0.96)	0.006	0.84 (0.71, 1.00)	0.055	0.61 (0.44, 0.86)	0.004
Water availability all year						
No	1		1		1	
Yes	0.77 (0.67, 0.89)	< 0.001	0.75 (0.60, 0.95)	0.016	0.41 (0.24, 0.68)	0.001
Child’s Age in months						
0–23 months	1		1		1	
24–59 months	0.93 (0.82, 1.06)	0.279	1.23 (1.00, 1.52)	0.047	1.48 (1.00, 2.18)	0.048
Sources of drinking water						
Improved	1		1		1	
Unimproved	1.30 (1.13, 1.50)	< 0.001	0.95 (0.75, 1.22)	0.701	0.42 (0.25, 0.71)	0.001
Diarrhoea						
No diarrhoea	1		1		1	
Had diarrhoea	1.18 (1.02, 1.37)	0.030	2.26 (1.83, 2.80)	< 0.001	3.66 (2.51, 5.32)	< 0.001

Confounding factors adjusted for are: Socio-economic and demographic factors; child factors; child undernutrition status, maternal, health services and environmental factors and child’s health factors

### Factors associated with mild, moderate and severe underweight

Table 4 shows the factors associated with mild, moderate and severe underweight in children aged 0–59 months in three disadvantaged East African districts. The odds of a child being mildly, moderately or severely underweight were significantly higher in Kitgum district compared with Kilindi and Gicumbi districts. Increased odds of mild, moderate and severe wasting were observed among mother of children who did not attend child monthly growth monitoring sessions, a child aged 0–23 months and children who reported having diarrhoea. Children who had ARI symptoms were 1.18 times and 1.82 times more likely to report higher odds of moderately or severely underweight, respectively.

**Table 4:** Factors associated with mildly, moderately and severely underweights in children aged 0–59 months in three disadvantaged East African Districts

Variables	Mildly underweight		Moderately underweight		Severely underweight	
	AOR (95%CI)	*P*-value	AOR (95%CI)	*P*-value	AOR (95%CI)	*P*-value
District (Country)						
Gicumbi (Rwanda)	1		1		1	
Kitgum (Uganda)	5.66 (4.48, 7.19)	< 0.001	11.64 (7.09, 19.11)	< 0.001	1.11 (0.60, 2.07)	0.741
Kilindi (Tanzania)	1.00 (0.76, 1.32)	0.997	1.93 (1.08, 3.47)	0.027	0.20 (0.10, 0.40)	< 0.001
Household wealth index						
Poorest	1		1		1	
Middle	0.92 (0.77, 1.11)	0.393	0.47 (0.33, 0.67)	< 0.001	1.65 (1.03, 2.66)	0.038
Least Poor	0.89 (0.74, 1.07)	0.211	1.25 (0.95, 1.64)	0.107	1.48 (0.92, 2.35)	0.103
Sex of the baby						
Male	1		1		1	
Female	1.30 (1.12, 1.50)	< 0.001	0.75 (0.59, 0.95)	0.018	1.31 (0.90, 1.91)	0.154
Attended child monthly growth monitoring sessions						
Yes	1		1		1	
No	1.11 (0.93, 1.32)	0.232	1.33 (1.02, 1.74)	0.036	2.19 (1.44, 3.35)	< 0.001
Child’s Age in months						
0–23 months	1		1		1	
24–59 months	0.50 (0.43, 0.59)	< 0.001	0.39 (0.30, 0.51)	< 0.001	0.30 (0.18, 0.49)	< 0.001
Sources of drinking water						
Improved	1		1		1	
Unimproved	1.12 (0.92, 1.36)	0.267	0.94 (0.65, 1.35)	0.727	3.28 (2.07, 5.19)	< 0.001
Type of toilet facility						
Improved	1		1		1	
Unimproved	2.29 (1.37, 3.85)	0.002	1.42 (0.73, 2.75)	0.302	0.59 (0.27, 1.31)	0.198
Diarrhoea						
No diarrhoea	1		1		1	
Had diarrhoea	1.21 (1.01, 1.44)	0.044	1.17 (0.87, 1.54)	0.267	8.90 (5.59, 14.17)	< 0.001
ARI						
No ARI	1.00		1.00		1.00	
Had ARI	0.69 (0.56,0.84)	< 0.001	1.18 (0.90,1.56)	0.235	1.87 (1.19,2.91)	0.006

Confounding factors adjusted for are: Socio-economic and demographic factors; child factors; child undernutrition status, maternal, health services and environmental factors and child’s health factors

## Discussion

This study identified the factors associated with childhood undernutrition in three East African Districts. The main factors associated with moderate or severe stunting/wasting were: wealth index (poorest households), increasing child’s age, sex of the child (male) and unavailability of water all year. Children of women who did not attend monthly child growth monitoring sessions and children who had ARI symptoms were significantly associated with moderate or severe underweight while having diarrhoea two weeks prior to the survey was significantly associated with severe undernutrition (stunting, wasting and underweight).

In this study, children under-five years residing in Gicumbi District in Rwanda were more predisposed to stunting while the odds of a child being wasted and underweight were higher in Kitgum District in Uganda. Though all three districts analysed were in East Africa, disparities still existed in the distribution of child undernutrition, and this might be due to specific features associated with the district’s geographic location. This report shows that geographic location may influence child nutrition and is a determinant of childhood undernutrition. Geographic location affects the cultivation of food crops, which in turn affects access and availability of food for household consumption and sale. The impact of geographical location could be seen in the environmental variability and predominant occupation practised in the region which could influence food (in) security and consequently affect child nutrition, growth and development [21, 22]. Geographic locations with more favourable climatic conditions and whose residents are predominantly farmers have greater access to variable food types and are more food secured [21]. However, studies have shown that cultural beliefs and practices unique to a region influence the food given to the growing child despite its overall nutritional value and availability [6, 7, 23].

In this study, the wealth index was used as a proxy to access socioeconomic status. Children from poor households were more susceptible to childhood stunting and wasting when compared to their counterparts from richer households in all three East African Districts. Evidence from previously conducted studies shows the inverse relationship between socioeconomic status and childhood stunting/wasting [6, 24–26]. These studies confirm that children from households with low socioeconomic status lack access to sufficient food of adequate quality, basic health care services, and have a higher risk of infection as a result of compromised immunity due to poor nutrition and suboptimal living conditions [27].

Child’s age was also reported as a major factor associated with stunting and wasting in the study area. Suboptimal growth increased with age as older children were reported to be more predisposed to childhood stunting/wasting. This increase in child undernutrition with age could be as a result of an increase in energy expenditure by the older child without sufficient and adequate food intake. It could also result from an increased interaction of the older child with its immediate environment which may lead to increased risk of infections and exposure to childhood diseases either through drinking water from unimproved sources, consumption of contaminated foods, poor hygiene or poor environmental sanitation [27]. Furthermore, as the child’s age increases, s/he requires more energy (calories) and nutrients for proper growth and development. The inability of a growing child to meet required daily energy and nutrient needs could result in undernourishment [7].

Male children were more prone to childhood stunting/wasting than their female counterparts. Male children tend to be more physically active thereby expending large amounts of energy which should be channelled towards proper growth and development. This finding is consistent with results from other studies carried out in Nigeria [6, 7], Iran [24], Kenya [28], Indonesia [29], Tanzania [30] Ghana [21], Ethiopia [31] and South Africa [32]. The studies reported a higher prevalence of childhood undernutrition among male children than females. However, a biological reason for this is still unknown.

Unavailability of water was reported as a significant factor associated with moderate and severe stunting. Lack of access to clean and safe water from improved water sources could have a significant detrimental effect on child growth and development resulting from sustained exposure to enteric pathogens and diarrhoeal disease [33]. Thus, improvements in the quantity and microbial quality of water available to the growing child is essential if the child is to meet its nutritional needs and reduce the risk of infection or dehydration. Additionally, the unavailability of water for irrigation purposes affects farming leading to low food production and food insecurity especially in locations with low rainfall. Household unavailability of water may result from the inability of households with low socio-economic status to afford the cost of clean drinking water. Therefore, to improve access to water and the nutritional status of children, consistent access to affordable, clean water, especially in disadvantaged communities, is vital [33].

Having diarrhoeal episodes and manifesting ARI symptoms two weeks prior to the survey were significantly associated with severe undernutrition. Studies have confirmed the relationship between ARI, diarrhoea and undernutrition which results in a vicious cycle [34–38]. Gastrointestinal and respiratory infections reduce appetite, increases catabolism and inhibit intestinal absorption of nutrients from food thus leading to increased susceptibility to severe undernutrition especially underweight [36, 37]. Consequently, child undernutrition leads to immune dysfunction, such as the impairment of cell-mediated immunity, cytokine and immunoglobulin production which lowers immunity and predisposes a child to infectious diseases [36, 38, 39]. Diarrhoea and ARI often occur due to suboptimal child feeding practices and an unhealthy environment [40]. Studies have also shown that exclusive breastfeeding in the first 6 months of life and the timely introduction of appropriate complementary foods (6–23 months) reduces the risk of diarrhoea and ARI [36, 37]. Proper child feeding practices build the child’s immunity through the transfer of innate immune components such as secretory IgA, lactoferrin and lysozyme from mother to child during breastfeeding and enhance antibody response to pathogens thus preventing infections [36, 37]. Therefore, to prevent ARI and the occurrence of diarrhoea, interventions targeted at promoting exclusive breastfeeding, vaccination, Los-osmolarity ORS, zinc and Vitamin A supplementation, availability of safe water, improved sanitation practices and preventing household pollution should be effected [40].

Children of women who did not attend monthly child growth monitoring sessions were significantly associated with moderate or severe underweight. Attending monthly child growth monitoring sessions allows for the evaluation of child growth in order to assess the appropriateness of the child’s nutrient intake and sanitation.

Additionally, the high prevalence of undernutrition in children under-5 years reported in this study may be attributed to the main lean period (October–December) before the survey when food prices are unreasonably high due to seasonal variability, and this continues up to harvest period before gradually decreasing.

### Strengths and limitations

This study had several strengths. First, the study was population-based with a large sample size which is representative of the study area. Second, it applied appropriate statistical adjustments to data obtained from the survey and identified the most vulnerable subpopulation affected by childhood undernutrition in a large sample. However, this study also had some limitations. First, due to the cross-sectional nature of the study design, a causal relationship between the observed risk factors and childhood undernutrition cannot be established. Second, this study did not include validity assessments of undernutrition; no actual dietary assessments were made. Third, despite the use of a comprehensive set of variables in the analysis, the effect of genetics, birth defects, and early food allergies as well as the effect of residual confounding as a result of unmeasured covariates were not addressed. Finally, the measure of household wealth index using a small number of variables may misrepresent household expenditure and the actually socioeconomic status of the household.

### Policy implications

Findings from this study are useful for public health planning to improve the nutritional status of children under-five years in East Africa. Intervention strategies geared towards reducing undernutrition in East African communities should focus on children from poor households with suboptimal sanitation practices. Community-based educational sessions which educate women on the benefits of attending postnatal monthly child growth monitoring sessions should also be initiated.

## Conclusions

Findings from our study indicate that having diarrhoea, ARI, unavailability of water supply all year and failure to attend monthly child growth monitoring sessions increases the odds of childhood undernutrition, especially among socioeconomically disadvantaged households. Thus interventions to reduce childhood undernutrition should focus on improving household sanitation and access to adequate water supply, as well as initiating community-based educational campaigns on the merits of attending child growth monitoring sessions. These interventions will improve the nutritional status of children under-five years in these East African communities which will invariably result in an overall decline in childhood undernutrition in Sub-Saharan Africa hereby setting the region on the path to achieving the WHO global nutrition target by 2025.

## Additional file

Additional file 1: Table S1: Percentage distribution of undernutrition in children aged 0-59 months by three East African Districts (*N*= 8,706). (DOCX 15 kb)

## Abbreviations

ANC: Antenatal Care
AOR: Adjusted Odd Ratios
ARI: Acute Respiratory Infection
CIs: Confidence Intervals
HIV: Human Immunodeficiency Virus
MNCH: Maternal Newborn Child Health
UNICEF: United Nations Children’s Fund
WHO: World Health Organization
