# Supplementary material for: Childhood undernutrition in three disadvantaged East African Districts: a multinomial analysis
Source: BMC Pediatr. 2019 Apr 23;19:118. doi: 10.1186/s12887-019-1482-y (PMC6477742; doi:10.1186/s12887-019-1482-y)
Supplement: Supplementary file 1 — Table S1: Percentage distribution of undernutrition in children aged 0-59 months by three East African Districts (N= 8,706). (DOCX 15 kb) [file 12887_2019_1482_MOESM1_ESM.docx]

Additional file 1: Table S1: Percentage distribution of undernutrition in children aged 0-59 months by three East African Districts (N= 8,706)

| **District (Country)** | Not | | | | Mildly | | | | Moderately | | | | Severely |
| --- | --- | --- | --- | --- | --- | --- | --- | --- | --- | --- | --- | --- | --- |
| **Stunting** | | | | | | | | | | | | | |
| Gicumbi (Rwanda) | | | 821 (9.4) | | | 543 (6.2) | | | | 513 (5.9) | | | 472 (5.4) |
| Kitgum (Uganda) | | | 1560 (17.9) | | | 1328 (15.3) | | | | 575 (6.6) | | | 240 (2.8) |
| Kilindi (Tanzania) | | | 1135 (13.0) | | | 534 (6.1) | | | | 543 (5.1) | | | 442 (5.1) |
| **Wasting** | | | | | | | | | | | | | |
| Gicumbi (Rwanda) | | 1867 (21.4) | | | | | 366 (4.2) | | | | 91 (1.0) | | 25 (0.3) |
| Kitgum (Uganda) | | 1959 (22.5) | | | | | 1260 (14.5) | | | | 383 (4.4) | | 101 (1.2) |
| Kilindi (Tanzania) | | 2241 (25.7) | | | | | 291 (3.3) | | | | 98 (1.1) | | 24 (0.3) |
| **Underweight** | | | | | | | | | | | | | |
| Gicumbi (Rwanda) | | | | 2162 (24.8) | | | | 130 91.5) | | | | 22 (0.3) | 35 (0.4) |
| Kitgum (Uganda) | | | | 2701 (31.0) | | | | 677 (7.8) | | | | 260 (3.0) | 65 (42.5) |
| Kilindi (Tanzania) | | | | 2401 (27.6) | | | | 178 (2.0) | | | | 39 (0.4) | 36 (0.4) |
